# Spotlight on the 2024 ESC/EACTS management of atrial fibrillation guidelines: 10 novel key aspects

**DOI:** 10.1093/europace/euae298

**Published:** 2024-12-24

**Authors:** Michiel Rienstra, Stylianos Tzeis, Karina V Bunting, Valeria Caso, Harry J G M Crijns, Tom J R De Potter, Prashanthan Sanders, Emma Svennberg, Ruben Casado-Arroyo, Jeremy Dwight, Luigina Guasti, Thorsten Hanke, Tiny Jaarsma, Maddalena Lettino, Maja-Lisa Løchen, R Thomas Lumbers, Bart Maesen, Inge Mølgaard, Giuseppe M C Rosano, Renate B Schnabel, Piotr Suwalski, Juan Tamargo, Otilia Tica, Vassil Traykov, Dipak Kotecha, Isabelle C Van Gelder

**Affiliations:** Department of Cardiology, University of Groningen, University Medical Center Groningen, P.O. Box 30.001, 9700 RB Groningen, The Netherlands; Department of Cardiology, Mitera Hospital, Athens, Greece; Institute of Cardiovascular Sciences, University of Birmingham, Birmingham, UK; Stroke Unit, Santa Maria della Misericordia-University of Perugia, Perugia, Italy; Department of Cardiology, Maastricht University Medical Center, Maastricht, The Netherlands; Department of Cardiology, Cardiovascular Research Institute Maastricht, Maastricht University, Maastricht, The Netherlands; Department of Cardiology, OLV Hospital, Aalst, Belgium; Centre for Heart Rhythm Disorders, University of Adelaide and Royal Adelaide Hospital, Adelaide, SA, Australia; Department of Medicine Karolinska University Hospital (MedH), Karolinska Institutet, Stockholm, Sweden; Department of Cardiology, H.U.B.-Hôpital Erasme, Université Libre de Bruxelles, Brussels, Belgium; ESC Patient Forum, Sophia Antipolis, France; Department of Medicine and Surgery, University of Insubria, Varese, Italy; Division of Geriatrics and Clinical Gerontology, ASST-Settelaghi, Varese, Italy; Clinic For Cardiac Surgery, Asklepios Klinikum, Harburg, Hamburg, Germany; Department of Cardiology, Linkoping University, Linkoping, Sweden; Julius Center for Health Sciences and Primary Care, University Medical Center Utrecht, Utrecht, The Netherlands; Department for Cardiac, Thoracic and Vascular Diseases, Fondazione IRCCS San Gerardo dei Tintori, Monza, Italy; Department of Clinical Medicine, UiT, The Arctic University of Norway, Tromsø, Norway; Department of Cardiology, University Hospital of North Norway, Tromsø, Norway; Institute of Health Informatics, University College London, London, UK; Saint Bartholomew’s Hospital, Barts Health NHS Trust, London, UK; University College Hospital, University College London Hospitals NHS Trust, London, UK; Department of Cardiothoracic Surgery, Maastricht University Medical Centre+, Maastricht, The Netherlands; Cardiovascular Research Institute Maastricht, Maastricht University, Maastricht, The Netherlands; ESC Patient Forum, Sophia Antipolis, France; Department of Human Sciences and Promotion of Quality of Life, Chair of Pharmacology, San Raffaele University of Rome, Rome, Italy; Department of Cardiology, San Raffaele Cassino Hospital, Cassino, Italy; Cardiovascular Academic Group, St George’s University Medical School, London, UK; Cardiology University Heart and Vascular Center Hamburg, University Medical Center Hamburg-Eppendorf, Hamburg, Germany; German Center for Cardiovascular Research (DZHK) Partner Site Hamburg/Kiel/Lübeck, Germany; Department of Cardiac Surgery and Transplantology, National Medical Institute of the Ministry of Interior and Administration, Centre of Postgraduate Medical Education, Warsaw, Poland; Pharmacology and Toxicology School of Medicine, Universidad Complutense, Madrid, Spain; Institute of Cardiovascular Sciences, University of Birmingham, Birmingham, UK; Department of Cardiology, Emergency County Clinical Hospital of Bihor, Oradea, Romania; Department of Invasive Electrophysiology, Acibadem City Clinic Tokuda University Hospital, Sofia, Bulgaria; Institute of Cardiovascular Sciences, University of Birmingham, Birmingham, UK; NIHR Birmingham Biomedical Research Centre, University Hospitals Birmingham NHS Foundation Trust, Birmingham, UK; Department of Cardiology, University of Groningen, University Medical Center Groningen, P.O. Box 30.001, 9700 RB Groningen, The Netherlands

**Keywords:** Atrial fibrillation, Management, Guidelines

## Abstract

Atrial fibrillation (AF) remains the most common cardiac arrhythmia worldwide and is associated with significant morbidity and mortality. The European Society of Cardiology (ESC)/European Association for Cardio-Thoracic Surgery (EACTS) have recently released the 2024 guidelines for the management of AF. This review highlights 10 novel aspects of the ESC/EACTS 2024 Guidelines. The AF-CARE framework is introduced, a structural approach that aims to improve patient care and outcomes, comprising of four pillars: [C] Comorbidity and risk factor management, [A] Avoid stroke and thromboembolism, [R] Reduce symptoms by rate and rhythm control, and [E] Evaluation and dynamic reassessment. Additionally, graphical patient pathways are provided to enhance clinical application. A significant shift is the new emphasis on comorbidity and risk factor control to reduce AF recurrence and progression. Individualized assessment of risk is suggested to guide the initiation of oral anticoagulation to prevent thromboembolism. New guidance is provided for anticoagulation in patients with trigger-induced and device-detected sub-clinical AF, ischaemic stroke despite anticoagulation, and the indications for percutaneous/surgical left atrial appendage exclusion. AF ablation is a first-line rhythm control option for suitable patients with paroxysmal AF, and in specific patients, rhythm control can improve prognosis. The AF duration threshold for early cardioversion was reduced from 48 to 24 h, and a wait-and-see approach for spontaneous conversion is advised to promote patient safety. Lastly, strong emphasis is given to optimize the implementation of AF guidelines in daily practice using a patient-centred, multidisciplinary and shared-care approach, with the simultaneous launch of a patient version of the guideline.

## Graphical Abstract

**Graphical Abstract:**
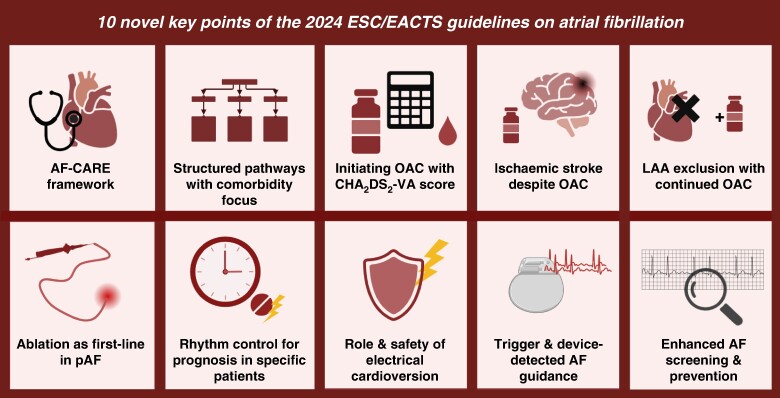
Overview of 10 novel key points in the 2024 ESC/EACTS Guidelines on the management of atrial fibrillation. AF, atrial fibrillation; LAA, left atrial appendage; OAC, oral anticoagulant; pAF, paroxysmal atrial fibrillation.

## Introduction

Despite significant advances in prevention, diagnosis, and treatment, atrial fibrillation (AF) remains common and continues to have a large impact on those living with AF, their relatives, and wider society.^1–3^ Guidelines for the management of AF intend to maintain a 4-year update cycle, with interim updates targeted every 2 years. The European Society of Cardiology (ESC)/European Association for Cardio-Thoracic Surgery (EACTS) 2024 AF guidelines aim to evaluate and summarize available evidence to assist health professionals in optimizing their diagnostic or therapeutic approach for individual patients with AF.^4^ The guideline was developed by a specifically assigned task force representing the ESC and EACTS, with contribution by the European Heart Rhythm Association (EHRA) and endorsement by the European Stroke Organisation. In this iteration of the AF guidelines, 130 recommendations are provided with underpinning evidence from robust clinical research, and a novel structured style is used for each recommendation to aid implementation. In addition, a patient version of the guideline was made available simultaneously (https://www.escardio.org/static-file/Escardio/Guidelines/Documents/ESC-Patient-Guidelines-Atrial-Fibrillation.pdf). This review aims to highlight 10 novel key aspects of the full ESC/EACTS 2024 Guidelines. Further details can be found in the full ESC/EACTS 2024 AF management guidelines.^4^

### Principles of AF-CARE approach

**Figure 1:**
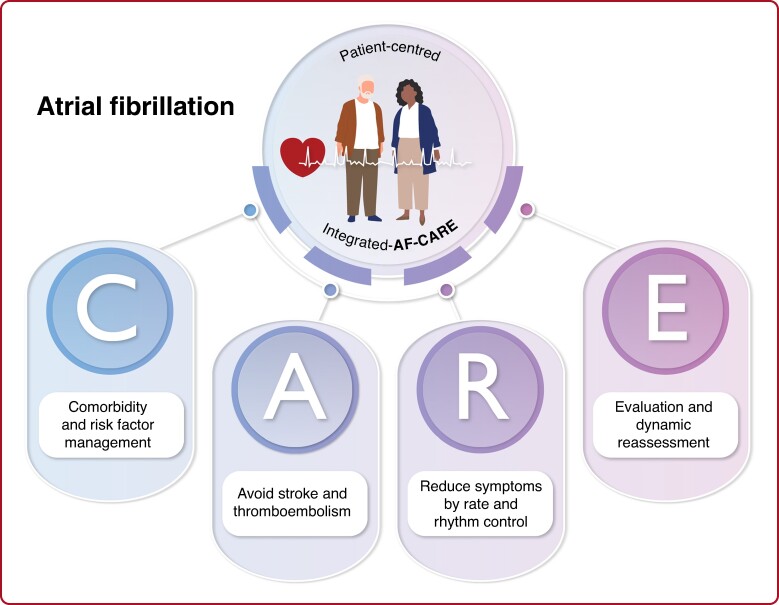
AF-CARE framework. AF, atrial fibrillation; CARE, [C] Comorbidity and risk factor management, [A] Avoid stroke and thromboembolism, [R] Reduce symptoms by rate and rhythm control, [E] Evaluation and dynamic reassessment. Adapted from EHJ 2024.^4^

The 2024 ESC/EACTS Guidelines on AF introduced the AF-CARE framework, a structured approach to AF management designed to enhance patient-centred care and outcomes.^4,5^ AF-CARE builds on previous frameworks,^6,7^ organizing care into four key pillars that integrate evidence-based management of AF with individualized patient needs (*Figure 1*). The pillars include: [C] *Comorbidity and risk factor management*, highlighting and bringing to the forefront the need for thorough evaluation and management of comorbidities and risk factors related to AF; [A] *Avoid stroke and thromboembolism*, prioritizing stroke and thromboembolism prevention through appropriate anticoagulation; [R] *Reduce symptoms by rate and rhythm control*, aiming for symptom relief and in specific patient groups adjunctive prognostic benefit; and [E] *Evaluation and dynamic reassessment,* emphasizing the need for a thorough baseline evaluation of patients with AF, including an echocardiogram for all patients with AF where this might guide treatment decisions, followed by continuous modification of care as patients living with AF and its associated comorbidities and risk factors evolve over time.

The systematic and patient-oriented AF-CARE framework serves as a guide that adapts with each patient, promoting a personalized and adaptive approach to AF management. By aligning care with the changing nature of AF and its associated comorbidities and risk factors, the wishes and needs of patients, and the continuous improvement of the management of AF, AF-CARE aims to improve outcomes and provide equal and optimal quality of care for all those encountering AF.

### New patient pathways for atrial fibrillation management with comorbidity and risk factor identification and management as a first step

The 2024 ESC/EACTS Guidelines present new structured patient pathways tailored to AF management based on AF temporal patterns—first-diagnosed, paroxysmal, persistent, and permanent. The pathways enhance the clinical application of the AF-CARE framework by providing clear, step-by-step guidance for individualized patient care, ensuring that management strategies can be easily adapted as a patient’s AF and (non-)cardiovascular status changes over time. An interactive mobile app enables a simple and portable approach for healthcare providers to access the 2024 guidelines and is provided free of charge (https://www.escardio.org/Guidelines/Clinical-Practice-Guidelines/Guidelines-derivativeproducts/ESC-Mobile-Pocket-Guidelines).

A significant shift in the 2024 guidelines is the emphasis on comorbidity and risk factor management. The guidelines set precise targets for managing AF-associated conditions and risk factors for all those comorbidities with a sufficient evidence base, such as hypertension, heart failure, diabetes, obesity, obstructive sleep apnoea, physical activity, and alcohol intake. Nevertheless, patients with AF may have other (non-)cardiovascular comorbidities that relate to AF and may impact management. These comorbidities and risk factors also require attention by patients and their healthcare professionals.^8–11^ New evidence demonstrates that effective management of comorbidities and risk factors can improve symptoms and quality of life, reduce AF recurrences, slow or prevent AF progression, improve the outcome of rhythm control strategies, and improve prognosis.^9–38^

By integrating these new comorbidity targets upfront into the patient pathways, the 2024 ESC/EACTS Guidelines reinforce that AF treatment strategies should not only be effective in controlling AF but also tailored to contribute to the broader health needs of each patient.

### Focus on providing oral anticoagulation using a locally validated risk score or the CHA_2_DS_2_-VA score

Atrial fibrillation significantly heightens the risk of thromboembolism, including ischaemic stroke, regardless of the temporal pattern of AF.^3,39,40^ Without appropriate treatment, the risk of ischaemic stroke in patients with AF is elevated five-fold, and one in every five strokes is linked to AF.^41^ Given this substantial risk, oral anticoagulation [preferably a direct oral anticoagulant (DOAC)] is advised for all eligible patients, except those at low risk of incident stroke or thromboembolism. The effectiveness of oral anticoagulation in preventing ischaemic stroke in patients with AF is well-documented.^42,43^ Antiplatelet therapies alone are not indicated for stroke prevention in AF.^44,45^

The 2024 ESC/EACTS Guidelines on AF introduced an important change in stroke risk assessment and oral anticoagulation initiation. An individualized approach to risk is advised, taking into account all potential thromboembolic risk factors. In the absence of locally validated risk scores, the CHA_2_DS_2_-VA score replaces the CHA_2_DS_2_-VASc score, with points assigned to the well-known stroke risk factors: congestive heart failure (one point), hypertension (one point), age ≥ 75 years (two points), diabetes mellitus (one point), prior stroke/transient ischaemic attack/arterial thromboembolism (two points), vascular disease (one point), and age 65–74 years (one point). Notably, the CHA_2_DS_2_-VA score omits consideration of gender, with the rationale that female sex does not contribute to decision making and is a risk modifier only in older patients that should already be receiving anticoagulation.^46–48^ In previous guidelines, separate recommendations were given for women and men, adding unnecessary complexity in clinical practice.^6^ The modification to CHA_2_DS_2_-VA seeks to simplify stroke risk assessment, with consistent thresholds regardless of gender.^49^

Oral anticoagulation using a DOAC, unless the patient has a mechanical valve or moderate–severe mitral stenosis, is advised for patients with AF with clearly elevated thromboembolic risk (CHA_2_DS_2_-VA score ≥2). Oral anticoagulation is advised to consider for patients with a CHA_2_DS_2_-VA score of 1. Physicians should explicitly discuss with their patients that decision making on anticoagulation treatment is dependent on the presence of stroke risk factors, or the presence of amyloidosis, hypertrophic cardiomyopathy, or mitral stenosis, and not on the type of AF or any rhythm control strategy employed. Despite maintenance of sinus rhythm or the absence of any AF-related symptoms, patients should continue oral anticoagulation based on the perceived risk of stroke and thromboembolism.^42,50–53^ Indeed, evidence demonstrates that there is no temporal relationship between ischaemic stroke occurrence and AF episodes.^54–56^

The 2024 ESC/EACTS Guidelines on AF also advocate for periodic reassessment of thromboembolic risk and attention to modifiable bleeding risk factors, to ensure that anticoagulation therapy remains appropriately aligned with each patient’s dynamic risk profile. These updates aim to enhance precision in stroke risk assessment and promote more widespread use of appropriate oral anticoagulation in patients with AF.^57–60^

### Ischaemic stroke despite anticoagulation

Oral anticoagulation is known to substantially reduce the risk of ischaemic stroke in patients with AF, yet a residual risk still remains.^61,62^ One-third of patients who suffer an ischaemic stroke are already on anticoagulation, which may be associated with factors including non-AF-associated stoke mechanisms, non-adherence, inadequate dosing, and ineffective anticoagulation.^63^ The 2024 ESC/EACTS Guidelines on AF introduced a new section to address these complex issues.

A thorough diagnostic work-up is advised for patients with AF who experience ischaemic stroke despite being on oral anticoagulation. This assessment should encompass a detailed and comprehensive evaluation of non-cardioembolic causes, vascular risk factors, medication dosage, and adherence to prevent recurrent events. Additionally, the guidelines explicitly advise against adding antiplatelet therapy to oral anticoagulation for preventing recurrent embolic stroke, due to an increased risk of bleeding, and no proven benefit.^64,65^ While switching from a vitamin K antagonist oral anticoagulant (VKA) to a DOAC may be beneficial for certain patient groups, the guidelines caution against routine switches from one DOAC to another, or from a DOAC to VKA without a clear indication, because these changes have not been demonstrated to be effective and may be harmful in some patients.^61,64,65^

The 2024 ESC/EACTS Guidelines on AF also emphasize avoiding a lower dose of DOAC, unless specific criteria are met (e.g. established renal dysfunction). Inappropriate dose reductions can heighten stroke risk without diminishing bleeding risks, underscoring the importance of adhering to full DOAC dosage to avoid preventable thromboembolism.^66–69^ New recommendations in the 2024 ESC/EACTS Guidelines on AF aim to enhance the management of patients with AF experiencing a stroke despite anticoagulation, with a focus on appropriate dosing and informed treatment decisions to improve patient outcomes.

### Left atrial appendage exclusion as an adjunct to oral anticoagulation in all concomitant, hybrid, or endoscopic surgery procedures

The left atrial appendage has long been recognized as a primary anatomical target for stroke prevention in patients with AF, as more than 90% of AF-related left atrial thrombi are located within it.^70^ The LAAOS III trial investigated the additive protective role of concomitant left atrial appendage occlusion in patients with AF undergoing cardiac surgery.^70^ During a 3-year follow-up, left atrial appendage occlusion significantly reduced ischaemic stroke or thromboembolism by one-third.^70^ Notably, several techniques were used for occlusion achievement (amputation with suture closing, stapling, or epicardial device closure) and 77% of patients with AF continued to receive oral anticoagulation at the end of the study. The incidence of safety events (perioperative bleeding, heart failure, or death) was similar between the compared groups.^71^ Based on the existing evidence, surgical closure of the left atrial appendage is recommended as an adjunct to oral anticoagulation in patients with AF undergoing cardiac surgery to prevent ischaemic stroke and thromboembolism.^70,72–76^

Left atrial appendage closure can also be performed during endoscopic or hybrid AF ablation with the use of external clip devices. Observational studies have demonstrated that left atrial appendage clipping during thoracoscopic AF ablation is feasible (95% complete left atrial appendage closure), safe (no intraoperative complications), and associated with a lower-than-expected rate of thromboembolism in patients maintaining post-procedural oral anticoagulation.^77^ Taking into account the existing non-randomized evidence,^78^ the 2024 ESC/EACTS Guidelines on AF support that any surgical closure of the left atrial appendage should be considered as an adjunct to oral anticoagulation in patients with AF undergoing endoscopic or hybrid AF ablation to prevent ischaemic stroke and thromboembolism.

### Catheter ablation as first-line rhythm control option in suitable patients with paroxysmal atrial fibrillation

Catheter ablation is a well-acknowledged invasive treatment for AF.^79^ Evidence-based credentials have established catheter ablation as the treatment of choice in patients with paroxysmal or persistent AF who are intolerant or resistant to anti-arrhythmic drugs.^80–83^ Based on accumulating evidence, the 2024 ESC/EACTS Guidelines on AF upgraded the role of catheter ablation as first-line rhythm control treatment option in anti-arrhythmic drug-naive patients with paroxysmal AF. This upgrade is supported by multiple randomized controlled trials, demonstrating that catheter ablation has superior efficacy and similar safety in comparison to anti-arrhythmic drugs regarding reduction of AF recurrences, alleviation of patient symptoms, improvement of quality of life, and delayed progression of AF.^84,85–93^

While catheter ablation is now a potential first option for maintaining sinus rhythm in patients with AF, this does not mean that every patient with paroxysmal AF should undergo catheter ablation. In essence, patients with paroxysmal AF should be informed about the possibility of catheter ablation, as part of the [R] pillar, after managing the [C] and [A] pillars. Every patient suitable for catheter ablation should be informed about the need for holistic AF treatment in the context of the entire AF-CARE pathway. Furthermore, every treatment decision in the management of patients with AF should be made together with each patient (shared decision making), considering the wishes and needs of each patient, and all potential treatment options in the context of respective risks and benefits. Note that ‘first-line’ is not synonymous with ‘first-time’, as several studies have demonstrated the unpredictable natural course of AF with one-quarter of patients presenting with no recurrence or a single AF recurrence during 3-year follow-up with continuous monitoring.^94^ The superiority of catheter ablation as first-line treatment in paroxysmal AF was demonstrated in randomized trials including patients with a substantial number of AF recurrences (e.g. median value of three symptomatic AF episodes per month in the EARLY-AF trial).^85^ Therefore, the pertinent favourable results should not be extrapolated to patients having experienced limited paroxysmal AF episodes.

### Rhythm control in selected patients can improve prognosis

Rhythm control is effective in alleviating AF-related symptoms and improving quality of life.^95,96^ In addition, in specific patient categories, sinus rhythm maintenance can also offer prognostic benefits. The 2024 ESC/EACTS Guidelines on AF have issued specific recommendations for rhythm control in these patient groups.

Implementation of a rhythm control strategy is advised to consider within 12 months of an AF diagnosis in selected patients with high risk of stroke or thromboembolism to reduce the risk of cardiovascular death or hospitalization, as evidenced by the Early treatment of Atrial fibrillation for Stroke prevention Trial (EAST-AFNET 4).^97,98^ It is crucial to consider that the characteristics of the enrolled patients in EAST-AFNET-4 (median duration since AF diagnosis 36 days, 54% in sinus rhythm and 30% asymptomatic at baseline) may not represent all patients with AF encountered in everyday practice.

Recent evidence has demonstrated that ablation may also confer benefits extending beyond symptom control in selected patient categories. Catheter ablation is advised in patients with AF and heart failure with reduced ejection fraction presumed to be due to tachycardia-induced cardiomyopathy, with the aim of reversing left ventricular dysfunction.^99,100^ Several clinical factors [New York Heart Association (NYHA) class, heart failure aetiology, and AF pattern] and imaging factors (left atrial dilatation and presence of atrial or ventricular fibrosis) may aid in the selection of suitability for catheter ablation.^101^ Furthermore, AF catheter ablation is advised to be considered in selected patients with AF and heart failure with reduced ejection fraction, where this could be expected to reduce heart failure hospitalization and mortality.^99,102–106^ Nevertheless, data on the prognostic benefit of catheter ablation in heart failure with reduced ejection fraction patients are not fully consistent, since there are also negative trials.^107,108^ Therefore, the 2024 ESC/EACTS Guidelines on AF emphasize the need for a patient-centred individualized approach in this patient group.

### Prioritize patient safety during cardioversion of atrial fibrillation

Cardioversion of AF is associated with a risk of stroke or thromboembolism in patients who have not received appropriate anticoagulation or where imaging has not excluded an intra-cardiac thrombus.^50,51,109,110^ This risk is variable and depends on patient characteristics and the duration of AF. The previously employed AF duration threshold of 48 h, which allowed early cardioversion without the need for anticoagulation or thrombus screening using transoesophageal echo, has been questioned by observational data.^111–113^ Furthermore, documentation of AF onset is dependent on patient self-reporting, and thus the reliable estimation of AF duration remains challenging. In the context of prioritizing safety, the 2024 ESC/EACTS Guidelines on AF recommend a shorter cut-off of 24 h known duration of AF for early cardioversion in patients who have not received at least 3 weeks of effective anticoagulation or thrombus exclusion with transoesophageal echocardiography.

Electrical cardioversion is highly effective in restoring sinus rhythm and is valuable in various clinical scenarios. In emergency settings, electrical cardioversion is recommended for patients with haemodynamic instability to improve immediate outcomes. Electrical cardioversion may also be used when the impact of AF-related symptomatology is not clear, or as a diagnostic tool when the benefits of restoring sinus rhythm are uncertain. The correlation between symptoms and heart rhythm is poor in patients with intermittent AF, since patient symptoms are not specific and may be due to coexistent comorbidities.^114^ A substantial percentage of patients without self-reported AF-related symptoms may experience improvement in their symptomatic status and functional class after electrical cardioversion.^115^ The diagnostic utility of electrical cardioversion may also prove helpful in patients with AF and impaired left ventricular function when AF-mediated tachycardiomyopathy is a differential diagnosis. In these cases, electrical cardioversion can assess the potential recovery of systolic function with sinus rhythm restoration.^115^ If AF is identified as the primary driver of systolic dysfunction, the patient could then be considered for catheter ablation.

Despite the utility of electrical cardioversion, spontaneous conversion to sinus rhythm is very likely in patients presenting with recent onset AF. In the RACE 7 ACWAS trial (Rate Control vs. Electrical Cardioversion Trial 7—Acute Cardioversion vs. Wait and See), comparing an early cardioversion with a wait-and-see approach, 69% of patients in the wait-and-see group (receiving only rate-control medications) experienced spontaneous recovery to sinus rhythm.^116^ Furthermore, the wait-and-see strategy was non-inferior to early cardioversion in maintaining sinus rhythm at 4 weeks, with more than 90% of patients in sinus rhythm. Therefore, a wait-and-see approach for spontaneous conversion to sinus rhythm should be considered as treatment option in shared decision making in patients without haemodynamic compromise as an alternative to immediate cardioversion. Regardless of approach and whether electrical or pharmacological cardioversion is used, it remains crucial that patients receive adequate therapeutic anticoagulation for at least 3 weeks before scheduled cardioversion, either by adherence to direct oral anticoagulants or consistent INR values >2 if using vitamin K antagonists.

### New guidance for trigger-induced and device-detected sub-clinical atrial fibrillation

Trigger-induced AF is a new AF episode in close proximity to a precipitating and potentially reversible factor. The most common precipitating factor for AF is sepsis, which is linked to an AF prevalence of 9–20%, and the chances of AF development increase with high degrees of inflammation.^117–120^ Other triggers include alcohol use, illicit drugs, and chronic inflammatory conditions. New in the 2024 guidelines, is advice to manage trigger-induced AF following the AF-CARE principles, emphasizing the need to address underlying reversible triggers and potential other comorbidities and risk factors. Long-term oral anticoagulation in patients with trigger-induced AF is advised to be considered according to perceived individual risk of stroke or thromboembolism, and started when potential increased bleeding risks related to certain acute triggers have been addressed. This advice reflects the observational evidence that these patients have similar AF recurrence rates, thromboembolic, and mortality risk as patients with clinical AF, although randomized trials are lacking in this context.^121–124^

New guidance has also been provided for device-detected sub-clinical AF (asymptomatic episodes of AF detected on continuous monitoring devices). The ARTESiA trial (Apixaban for the Reduction of Thromboembolism in Patients With Device-Detected Sub-Clinical Atrial Fibrillation) demonstrated that apixaban reduced the risk of stroke or systemic embolism compared with aspirin, although it also increased major bleeding risk.^125^ The NOAH trial (non-vitamin K antagonist oral anticoagulants in patients with atrial high rate episodes), which examined edoxaban compared with placebo, was terminated early due to futility and safety concerns, and found increased bleeding risk without associated efficacy.^126^ With the currently available evidence, direct oral anticoagulants may be considered for patients with device-detected sub-clinical AF, high stroke risk, and low bleeding risk, acknowledging the high chance of progression to clinical AF (6–9% per year), but also the bleeding risk accompanying anticoagulation. Whether a threshold exists based on certain duration of AF (episodes or burden) is still unclear.^127^

### Expanded strategies for screening and early detection of atrial fibrillation

Atrial fibrillation is the most common sustained arrhythmia worldwide and yet AF often remains undetected; its prevalence is expected to rise due to population growth, ageing, and improved survival from other cardiac conditions.^128,129^ The guidelines provide expanded approaches to population-based screening, early detection, and primary prevention of AF.

Population-based screening of AF through systematic programmes is now clearly distinguished from opportunistic detection during routine healthcare visits. Routine heart rhythm assessment during healthcare contact is recommended for all individuals aged ≥65 years to facilitate earlier detection of AF.^130,131^ Population-based screening using a prolonged non-invasive ECG-based approach in patients with AF aged ≥75 years or ≥65 years with additional CHA_2_DS_2_-VA risk factors should be considered to ensure timely detection of AF.^132–135^ The evidence on refinement of potential screening target populations, optimal screening durations, utility of new diagnostic and consumer wearable technologies, as well as the overall cost-effectiveness of routine and population-based screening is still too sparse to provide clear guidance.^95,136^

## Summary and conclusions

The recently published 2024 ESC/EACTS Guidelines on AF provide a comprehensive update of the evidence-based recommendations for optimal contemporary management of AF.^4^

One of the most significant changes is the introduction of the AF-CARE framework. Building on previous approaches, placing [C] Comorbidity and risk factor management at the forefront is a major shift. The proposed benefits are that better comorbidity and risk factor management will substantially contribute to improvement of symptoms and quality of life, reduction of AF recurrences, prevention of AF progression, enhanced effectiveness of rhythm control strategies, and lead to improvement in prognosis.

Concerning [A] Avoidance of stroke and thromboembolism, the new guidelines prioritize prevention through the appropriate use of oral anticoagulation based on locally validated risk scores or the CHA_2_DS_2_-VA score (using the same treatment cut-off regardless of gender). In addition, guidance is provided on trigger-induced AF, device-detected sub-clinical AF, and approaches to reduce the residual risk of stroke despite anticoagulation. The role of left atrial appendage occlusion needs further study, but is now indicated in all endoscopic, hybrid, or concomitant cardiac surgery procedures as an adjunct to oral anticoagulation.

Substantial evidence updates have enabled more targeted approaches to [R] Reduce symptoms by rate and rhythm control, necessitating a shared decision-making approach between patients and their multidisciplinary healthcare professionals. Catheter ablation of AF has been upgraded to a first-line rhythm control option in patients with paroxysmal AF in addition to those failing anti-arrhythmic drug therapy. The 2024 ESC/EACTS Guidelines on AF also provide guidance to improve prognosis with rhythm control in selected patients and more detailed information on how to safely perform cardioversion of AF.

Finally, the addition of [E] Evaluation and dynamic reassessment is an important and constructive step to ensure better provision of lifelong, optimal AF management, including identification and timely treatment of changing individual risk factors to prevent progression and adverse outcomes related to AF.

The 2024 ESC/EACTS Guidelines on AF have introduced patient flow charts that cover the major aspects of AF-CARE, using a consistent writing style for all recommendations (the intervention proposed, the population it is applied to, and the potential value to the patient, followed by any exceptions). Making the 2024 ESC/EACTS Guidelines on AF easier to read and more simple to follow will hopefully lead to better implementation. As evidenced by the ESC’s first randomized trial (STEEER-AF), delivering guideline-adherent management is critical if we are to reduce patient, health care, and societal burdens of AF,^137^ with a key role for the education of patients, carers, and healthcare professionals.^4,138^ All recommendations in the 2024 ESC/EACTS Guidelines on AF were supported by detailed supplementary evidence tables to provide a clear insight into all available evidence, limitations, and research gaps. The patient representatives of the 2024 ESC/EACTS Guidelines on AF also designed a patient version of the guidelines, to inform and empower patients with AF about their specific management options, with the goal to improve engagement and self-management of AF, and to facilitate optimal shared decision making.^139,140^

## Supplementary Material

euae298_Supplementary_Data

## Data Availability

No datasets were generated or analysed during the current study.
