# Supplementary material for: Spotlight on the 2024 ESC/EACTS management of atrial fibrillation guidelines: 10 novel key aspects
Source: Europace. 2024 Dec 24;26(12):euae298. doi: 10.1093/europace/euae298 (PMC11666470; doi:10.1093/europace/euae298)
Supplement: euae298_Supplementary_Data [file euae298_supplementary_data.zip › 2024-11-13_220440.pdf]

R. Thomas Lumbers	I agree to the new authorship list and contributions shown above in section 3, for the reasons outlined in section 2.		
Bart Maesen	I agree to the new authorship list and contributions shown above in section 3, for the reasons outlined in section 2.		
Inge Mølgaard	I agree to the new authorship list and contributions shown above in section 3, for the reasons outlined in section 2.	Inge Mølgaard	13.11.2024
Giuseppe M.C. Rosano	I agree to the new authorship list and contributions shown above in section 3, for the reasons outlined in section 2.		
Renate B. Schnabel	I agree to the new authorship list and contributions shown above in section 3, for the reasons outlined in section 2.		
Piotr Suwalski	I agree to the new authorship list and contributions shown above in section 3, for the reasons outlined in section 2.		
Juan Tamargo	I agree to the new authorship list and contributions shown above in section 3, for the reasons outlined in section 2.		
Otilia Tica	I agree to the new authorship list and contributions shown above in section 3, for the reasons outlined in section 2.		
Vassil Traykov	I agree to the new authorship list and contributions shown above in section 3, for the reasons outlined in section 2.		
Dipak Kotecha	I agree to the new authorship list and		
