# Supplementary material for: Spotlight on the 2024 ESC/EACTS management of atrial fibrillation guidelines: 10 novel key aspects
Source: Europace. 2024 Dec 24;26(12):euae298. doi: 10.1093/europace/euae298 (PMC11666470; doi:10.1093/europace/euae298)
Supplement: euae298_Supplementary_Data [file euae298_supplementary_data.zip › Change of authorship EuropaceWetSignature.pdf]

Europace

Change of Authorship Request Form

Title of manuscript: **Spotlight on the 2024 ESC/EACTS management of atrial fibrillation guidelines: 10 novel key aspects**

Submission Number: **EUPC-D-24-01087R1**

1. Previous Authorship List, in the order shown on the manuscript. Please indicate the corresponding author with a *

   <u>Authors:</u>
   Michiel Rienstra (Netherlands)* [#1], Stylianos Tzeis (Greece) [#2], Karina V. Bunting (United Kingdom)[3], Valeria Caso (Italy)[4], Harry J.G.M. Crijns (Netherlands)[5], Tom J.R. De Potter (Belgium)[6], Prashanthan Sanders (Australia)[7], Emma Svennberg (Sweden)[8], Dipak Kotecha (United Kingdom)[1,9], Isabelle C. Van Gelder (Netherlands)[1]

   [#] These authors contributed equally to this work.

   <u>Collaborators:</u>
   Ruben Casado-Arroyo (Belgium)[10], Jeremy Dwight (United Kingdom)[11], Luigina Guasti (Italy) [12], Thorsten Hanke (Germany) [13], Tiny Jaarsma (Sweden) [14], Maddalena Lettino (Italy) [15], Maja-Lisa Løchen (Norway) [16], R. Thomas Lumbers (United Kingdom) [17], Bart Maesen (Netherlands) [18], Inge Mølgaard (Denmark) [19], Giuseppe M.C. Rosano (United Kingdom) [20], Renate B. Schnabel (Germany) [21], Piotr Suwalski (Poland) [22], Juan Tamargo (Spain) [23], Otilia Tica (Romania) [24], Vassil Traykov (Bulgaria) [25]

2. Please provide an explanation for the change in authorship: (including any reasons for removal from authorship list)

   As per request of the CPG review performed via Europace editor, because paper is based on work of Task Force.

3. Proposed new authorship list (including email addresses), in the order it should appear on the manuscript. Please indicate the corresponding author with a *

Authors:

Michiel Rienstra (Netherlands)*[#1], Stylianos Tzeis (Greece)[#2], Karina V. Bunting (United Kingdom)[3], Valeria Caso (Italy)[4], Harry J.G.M. Crijns (Netherlands)[5], Tom J.R. De Potter (Belgium)[6], Prashanthan Sanders (Australia)[7], Emma Svennberg (Sweden)[8], Ruben Casado-Arroyo (Belgium)[9], Jeremy Dwight (United Kingdom)[10], Luigina Guasti (Italy)[11], Thorsten Hanke (Germany)[12], Tiny Jaarsma (Sweden)[13], Maddalena Lettino (Italy)[14], Maja-Lisa Løchen (Norway)[15], R. Thomas Lumbers (United Kingdom)[16], Bart Maesen (Netherlands)[17], Inge Mølgaard (Denmark)[10], Giuseppe M.C. Rosano (United Kingdom)[18], Renate B. Schnabel (Germany)[19], Piotr Suwalski (Poland)[20], Juan Tamargo (Spain)[21], Otilia Tica (Romania)[22], Vassil Traykov (Bulgaria)[23], Dipak Kotecha (United Kingdom)[3,24], Isabelle C. Van Gelder (Netherlands)[1]

[#] These authors contributed equally to this work.

All individuals listed as authors qualify for authorship and should have participated sufficiently in the work to take public responsibility for appropriate portions of the content. Each author fullfills all required conditions, as specified in the guidelines of the International Committee of Medical Journal Editors (ICMJE).

Michiel Rienstra	Groningen	Netherlands	m.rienstra@umcg.nl
Stylianos Tzeis	Athens	Greece	stzeis@otenet.gr
Karina V. Bunting	Birmingham	United Kingdom	k.v.bunting@bham.ac.uk
Valerio Caso	Perugia	Italy	vcaso@hotmail.com
Harry J.G.M. Crijns	Maastricht	Netherlands	hjgm.crijns@mumc.nl
Tom J.R. De Potter	Aalst	Belgium	tomdepotter@gmail.com
Prashanthan Sanders	Adelaide	Australia	prash.sanders@adelaide.edu.au
Emma Svennberg	Stockholm	Sweden	emma.svennberg@sll.se
Ruben Casado-Arroyo	Brussels	Belgium	ruben.casado.arroyo@erasme.ulb.ac.be
Jeremy Dwight		United Kingdom	jeremydwight@hotmail.com
Luigina Guasti	Varese	Italy	Luigina.Guasti@uninsubria.it
Thorsten Hanke	Hamburg	Germany	thorstenhanke@yahoo.com
Tiny Jaarsma	Linkoping	Sweden	tiny.jaarsma@liu.se
Maddalena Lettino	Monza	Italy	maddalena.lettino@hotmail.it
Maja-Lisa Løchen	Tromsø	Norway	maja-lisa.lochen@uit.no
R. Thomas Lumbers	London	United Kingdom	t.lumbers@ucl.ac.uk
Bart Maesen	Maastricht	Netherlands	maesen.bart@gmail.com
Inge Mølgaard		Denmark	molgaard.inge@gmail.com

Giuseppe M.C. Rosano	London	United Kingdom	giuseppe.rosano@gmail.com
Renate B. Schnabel	Hamburg	Germany	r.schnabel@uke.de
Piotr Suwalski	Warsaw	Poland	suwalski.piotr@gmail.com
Juan Tamargo	Madrid	Spain	jtamargo@med.ucm.es
Otilia Tica	Oradea	Romania	otilia.tica@gmail.com
Vassil Traykov	Sofia	Bulgaria	vtraykov@yahoo.com
Dipak Kotecha	Birmingham	United Kingdom	d.kotecha@bham.ac.uk
Isabelle van Gelder	Groningen	Netherlands	i.c.van.gelder@umcg.nl

4. All authors, unchanged, new, and removed must sign this declaration.

Signatures can be in the form of docu-sign, or handwritten signatures can be returned as an image file.

Typed names in the signature box WILL NOT be accepted.

Author name (first name, last name)	Declaration	Signature	Date signed
**Michiel Rienstra**	**I agree to the new authorship list and contributions shown above in section 3, for the reasons outlined in section 2.**		
**Stylianos Tzeis**	**I agree to the new authorship list and contributions shown above in section 3, for the reasons outlined in section 2.**		
**Karina V. Bunting**	**I agree to the new authorship list and contributions shown above in section 3, for the reasons outlined in section 2.**		
**Valeria Caso**	**I agree to the new authorship list and contributions shown above in section 3, for the reasons outlined in section 2.**		
**Harry J.G.M. Crijns**	**I agree to the new authorship list and contributions shown above in section 3, for the reasons outlined in section 2.**		
**Tom J.R. De Potter**	**I agree to the new authorship list and contributions shown above in section 3, for**		

	**the reasons outlined in section 2.**		
**Prashanthan Sanders**	**I agree to the new authorship list and contributions shown above in section 3, for the reasons outlined in section 2.**		
**Emma Svennberg**	**I agree to the new authorship list and contributions shown above in section 3, for the reasons outlined in section 2.**		
**Ruben Casado-Arroyo**	**I agree to the new authorship list and contributions shown above in section 3, for the reasons outlined in section 2.**		
**Jeremy Dwight**	**I agree to the new authorship list and contributions shown above in section 3, for the reasons outlined in section 2.**		
**Luigina Guasti**	**I agree to the new authorship list and contributions shown above in section 3, for the reasons outlined in section 2.**		
**Thorsten Hanke**	**I agree to the new authorship list and contributions shown above in section 3, for the reasons outlined in section 2.**		
**Tiny Jaarsma**	**I agree to the new authorship list and contributions shown above in section 3, for the reasons outlined in section 2.**		
**Maddalena Lettino**	**I agree to the new authorship list and contributions shown above in section 3, for the reasons outlined in section 2.**		
**Maja-Lisa Løchen**	**I agree to the new authorship list and contributions shown above in section 3, for the reasons outlined in section 2.**		

R. Thomas Lumbers	I agree to the new authorship list and contributions shown above in section 3, for the reasons outlined in section 2.		
Bart Maesen	I agree to the new authorship list and contributions shown above in section 3, for the reasons outlined in section 2.		
Inge Mølgaard	I agree to the new authorship list and contributions shown above in section 3, for the reasons outlined in section 2.		
Giuseppe M.C. Rosano	I agree to the new authorship list and contributions shown above in section 3, for the reasons outlined in section 2.		
Renate B. Schnabel	I agree to the new authorship list and contributions shown above in section 3, for the reasons outlined in section 2.		
Piotr Suwalski	I agree to the new authorship list and contributions shown above in section 3, for the reasons outlined in section 2.		
Juan Tamargo	I agree to the new authorship list and contributions shown above in section 3, for the reasons outlined in section 2.		
Otilia Tica	I agree to the new authorship list and contributions shown above in section 3, for the reasons outlined in section 2.		
Vassil Traykov	I agree to the new authorship list and contributions shown above in section 3, for the reasons outlined in section 2.		13.11.2024
Dipak Kotecha	I agree to the new authorship list and		

	**contributions shown above in section 3, for the reasons outlined in section 2.**		
**Isabelle C. Van Gelder**	**I agree to the new authorship list and contributions shown above in section 3, for the reasons outlined in section 2.**		
